# Minimally Invasive Resection of Benign Esophageal Lesions

**DOI:** 10.1053/j.optechstcvs.2014.12.002

**Published:** 2015-01-03

**Authors:** Ryan A. Macke, Katie S. Nason

**Affiliations:** 1 University of Wisconsin School of Medicine and Public Health, Department of Surgery, Madison, WI; 2 University of Pittsburgh, Department of Cardiothoracic Surgery, Division of Thoracic and Foregut Surgery, Pittsburgh, PA

**Keywords:** Minimally invasive surgery, Esophageal surgery, Benign esophageal disease, Enucleation, Leiomyoma

## Abstract

Benign esophageal lesions include a wide variety of rare neoplasms, polyps, and cysts. In general, these lesions are asymptomatic and have little clinical importance. However, on occasion these lesions become symptomatic due to esophageal obstruction, airway obstruction, or compression of mediastinal structures. In these cases, as well as cases when it is unclear if the lesion is malignant or benign, surgical resection is recommended. Resection is most often performed by extramucosal enucleation, a procedure that is oftentimes well-suited for a minimally invasive approach. Here we discuss the general approach and operative techniques used for minimally invasive resection of benign esophageal lesions.

## INTRODUCTION

Benign esophageal lesions are a heterogeneous group of rare neoplasms, polyps, and cysts. It is estimated that benign tumors represent less than 1% of all esophageal neoplasms and less than 10% of all resected esophageal neoplasms.^1^ The majority are located in the mid- or distal esophagus and can arise from any layer of the esophageal wall. Therefore, lesions are most often described as intraluminal, submucosal, intramural, or extraesophageal in origin.^2^ Intraluminal lesions arising from the mucosa include various polyps, papillomas, and inflammatory pseudotumors. Submucosal and intramural neoplasms include leiomyomas, granular cell tumors, hemangiomas, neurofibromas, lipomas, schwannomas, and rhabdomyomas. Leiomyomas are by far the most common histologic cell type, accounting for more than 80% of all benign esophageal tumors.^3^ Cysts, including intramural inclusions cysts and extraesophageal congenital duplication cysts, represent the second most common benign esophageal lesion.^4^ Gastrointestinal stromal tumors (GISTs) may be found anywhere in the gastrointestinal tract, including the esophagus. These neoplasms have malignant potential and management of these lesions is beyond the scope of this discussion.

Most benign esophageal neoplasms are asymptomatic, in part because of their indolent rate of growth and the ability of the esophagus to dilate. Because of this, the true incidence remains unknown. Most symptoms are caused by obstruction of the esophageal lumen or compression of surrounding mediastinal structures. Intraluminal lesions and large submucosal or intramural lesions, typically > 5 cm, are most likely to be symptomatic.^4^ As a result, dysphagia is the most common complaint on initial presentation. Other reported symptoms include chest pain, dyspnea, chronic cough, wheezing, and upper gastrointestinal bleeding.^4^ Circumferential growth of benign esophageal tumors can have the typical appearance and presentation of an esophageal stricture or malignant tumor.

Because dysphagia is the most common initial complaint, many patients undergo a contrast esophagram as part of the initial workup. This study will identify most symptomatic lesions, most often revealing a smooth-surfaced filling defect. Computed tomography scans are not typically necessary, but may be helpful in determining the degree of mass effect caused by the lesion on the esophagus and/or other mediastinal structures, as well as identifying other extraesophageal causes of symptoms. Upper endoscopy is important for confirmation of the diagnosis and for operative planning. Intraluminal lesions can be differentiated from lesions originating from deeper layers of the esophagus, which will have an intact layer of mucosa overlying the lesion. Endoscopic biopsy of intraluminal or submucosal lesions is recommended if there is any suspicion of malignancy. Biopsy is not recommended for intramural lesions, as adequate tissue is rarely obtained to rule out malignant disease (i.e. leiomyoma versus leiomyosarcoma)^2^ and the biopsy can create mucosal scarring that may increase the risk of perforation during enucleation.^3^ Establishing the location of the lesion relative to the cricopharyngeus and gastroesophageal junction is necessary to determine the optimal surgical approach (cervical, transthoracic, or transabdominal). Endoscopic ultrasound can be useful for assessing which layers of the esophageal wall are involved and evaluate for evidence of invasion into surrounding layers, which would raise suspicion for a malignant lesion.

Incidentally encountered lesions with minimal or no symptoms are typically observed. Symptomatic lesions (typically >5 cm) and lesions that are clearly growing warrant consideration of removal, particularly if there is concern for malignant transformation or to facilitate other esophageal procedures. Smaller intraluminal lesions can typically be resected with endoscopic techniques, such as EMR or polypectomy snaring for pedunculated lesions. Larger lesions may require resection via an esophagotomy. Submucosal, intramural, and extraesophageal lesions are typically amenable to extramucosal enucleation. Resection can be performed with low morbidity using traditional open techniques (thoracotomy and laparotomy);^5^ however the safety, feasibility, and improved outcomes associated with minimally invasive approaches (VATS and laparoscopy) are well documented and is preferred over open approaches in the hands of surgeons with expertise in minimally invasive esophageal surgery.^3^ There are no strict contraindications to a minimally invasive approach for the enucleation of benign lesions. However, lesion size, previous chest or abdominal surgery, prior biopsy, and surgeon experience all contribute to the technical difficulty of the procedure and should be considered when deciding between open or minimally invasive approach. Symptoms suggesting other esophageal pathology, such as reflux disease, hiatal hernia, and motility disorders should be appropriately worked up and addressed at the time of operation.

Given that the majority of benign esophageal lesions are submucosal or intramural in origin, the remaining discussion and illustrations will focus on the operative techniques used for minimally invasive extramucosal enucleation of these lesions.

## Conclusions

The authors typically avoid placing a nasogastric tube at the conclusion of the procedure. A contrast esophagram is obtained on postoperative day 1, assuming the patient is not nauseated and has a good, strong cough. Once esophageal leak has been ruled out, the patient is started on a clear liquid diet. The patient is discharged home with liquid oral pain medication and a bowel regimen once tolerating clear liquids. The diet is advanced gradually as an outpatient, starting with 3 days of clear liquids, followed by 3 days of full liquids, and finally soft solids. The patient is seen 1–2 weeks postoperatively in the clinic, at which time the patient is advanced to a regular diet if doing well.

No clear guidelines exist for long-term follow up of patients following enucleation of benign esophageal lesions. Follow up with repeat contrast esophagram in 6 months to a year postoperatively may be considered in cases where repair of mucosal perforation was needed or in cases of postoperative esophageal leak to monitor for esophageal structuring. Patients who develop recurrent symptoms should also be re-evaluated with a contrast esophagram and upper endoscopy to evaluate for late complications and new or recurrent lesions.

In summary, benign esophageal lesion enucleation is a procedure that is ideally suited for minimally invasive surgical techniques. Appropriate patient selection, work-up, approach, and port placement is key to a successful operation. Risk of mortality and morbidity is low, with mucosal perforation being the most feared perioperative complication.^3^ However, meticulous surgical technique and diligent investigation following enucleation should allow the surgeon to identify and repair the injury with minimal risk of postoperative morbidity. As always, conversion to an open operation should be undertaken without hesitation if necessary to fully achieve the goals of the operation and to ensure patient safety and excellent outcomes.

## Figures and Tables

**Figure 1:**
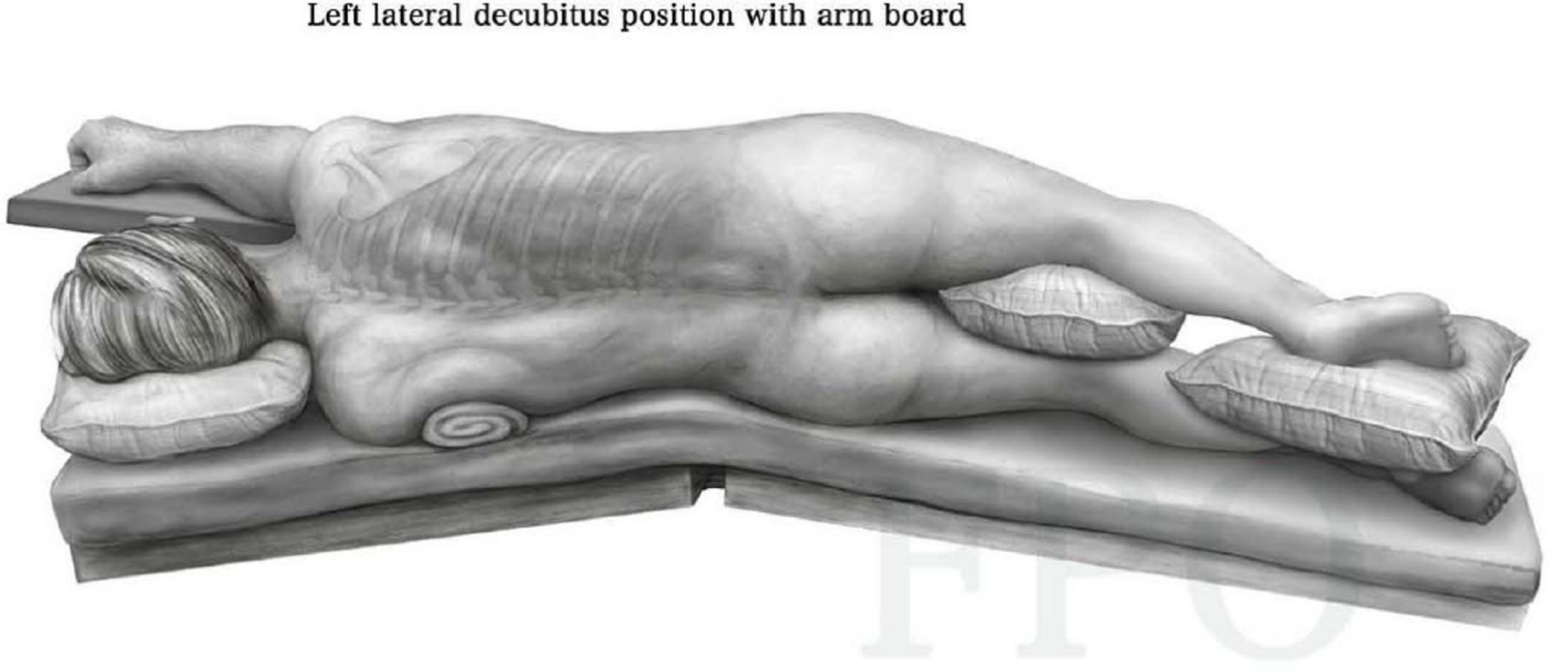
The right chest provides access to the esophagus from the level of the thoracic inlet to the diaphragm and is the authors’ preferred approach for most lesions more proximal than a few cm’s from the gastroesophageal junction. A similar left-sided approach may also be used for lesions located in the mid- or distal esophagus. The patient is positioned in the left lateral decubitus position with the right side up. The break in the operative table should be just above the superior aspect of the iliac crest to permit maximal flexion, thus, opening of the intercostal spaces. An axillary roll is placed 2–3 cm’s below the axilla. A beanbag allows maintenance of proper positioning throughout the procedure. The right arm is secured to an arm board and padded with the elbow and shoulder at approximately 90 degrees of flexion, which permits full range of motion of the instruments while preventing excessive stretch on the brachial plexus. Straps across the hips and legs with pillows at the knees and ankles secure the lower extremities. The operating table is rotated toward the patient’s front to allow the lung to fall anteriorly.

**Figure 2:**
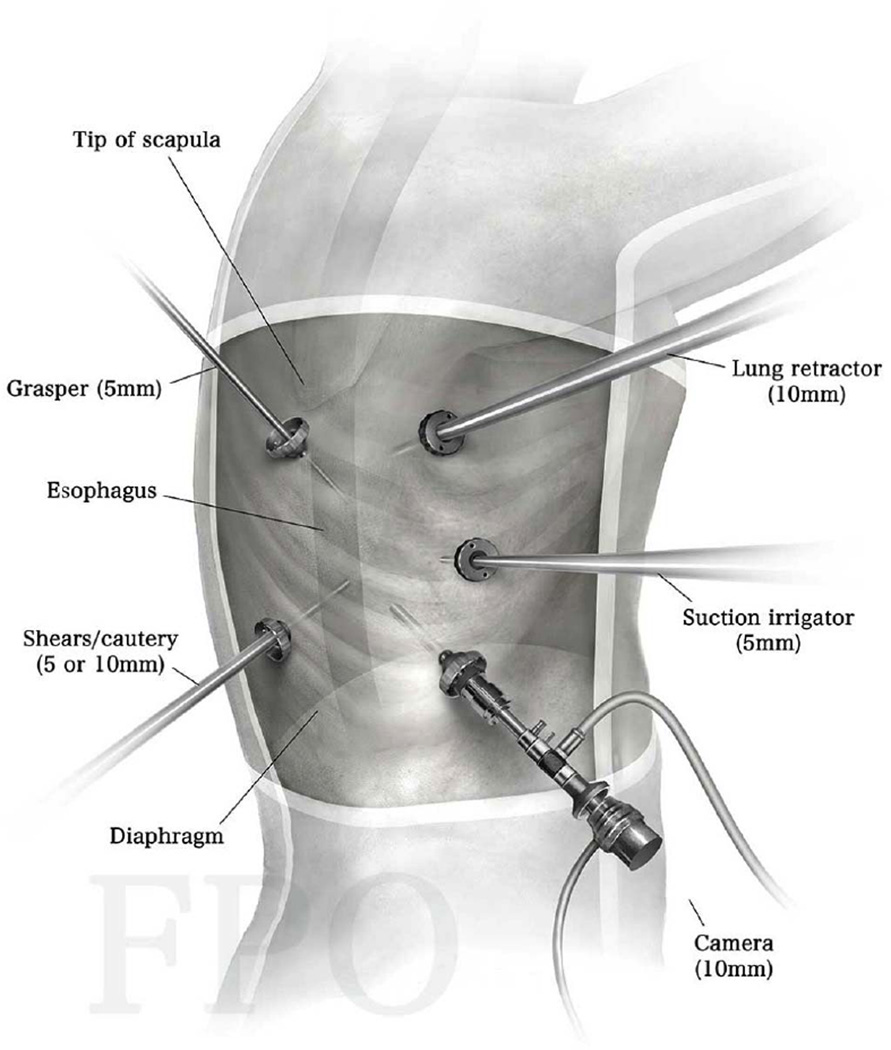
The operating surgeon stands on the right side of the table, facing the patient’s back. The assistant stands on the left side of the table, facing the patient’s front. Five thoracoscopic ports are placed. The 5 or 10 mm camera port is placed first, located in the posterior axillary line in the 7^th^ or 8^th^ intercostal space. The remaining ports are placed under direct thoracoscopic vision using a 30-degree scope. A 5 or 10 mm port is placed in line with the tip of the scapula posteriorly in the 7^th^ or 8^th^ intercostal space and serves as the surgeon’s right-handed working port. A 5 mm port is placed one intercostal space below the tip of the scapula for the surgeon’s left-handed working port. A 10 mm port is placed in the 4^th^ intercostal space in the mid-axillary line for the lung retractor. If a second assistant is available, an additional 5 mm port can be placed in the 6^th^ intercostal for an additional retraction port or for a suction-irrigator.

**Figure 3:**
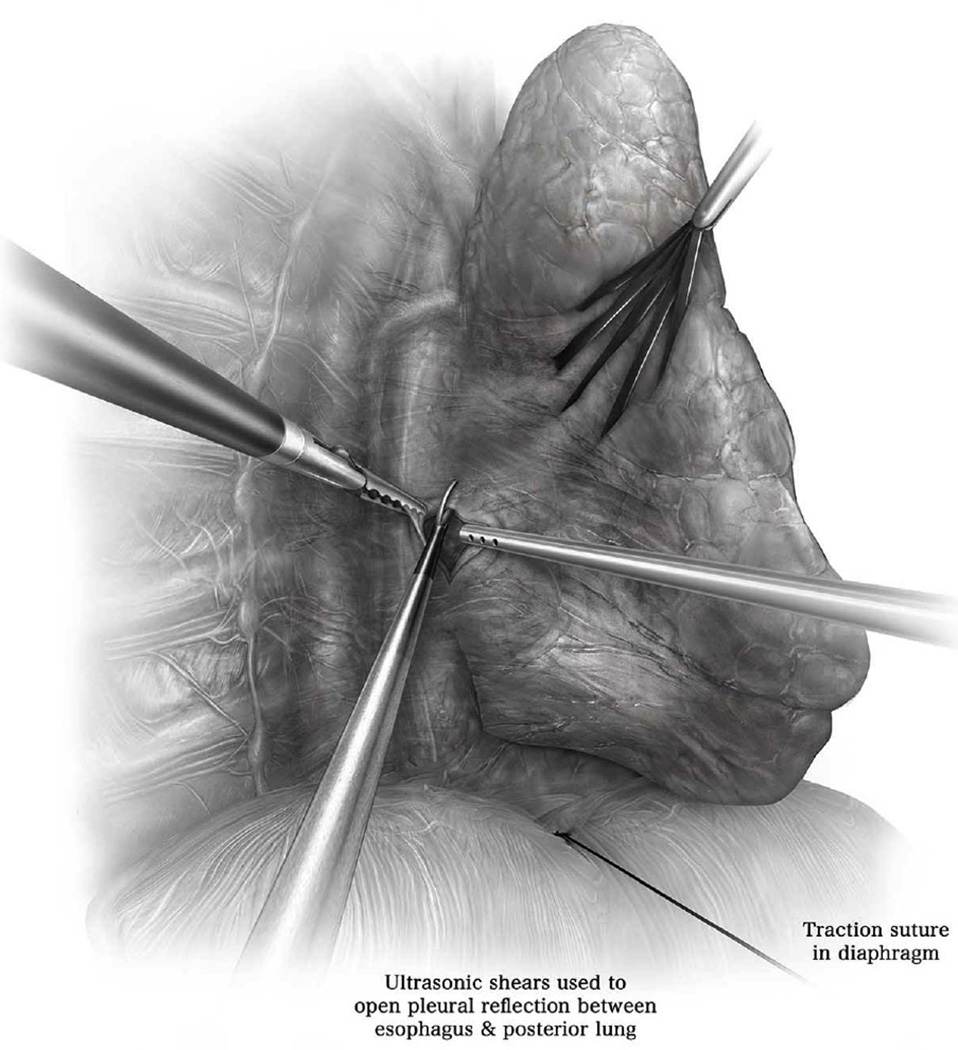
Once the inferior pulmonary ligament has been divided, the lung is retracted anteriorly. A diaphragm retraction stitch can be placed in the central tendon of the diaphragm to pull the diaphragm inferiorly, providing better visualization of the distal esophagus if needed. The stitch is brought through the chest wall anteriorly at the level of the diaphragm insertion. The mediastinal pleura overlying the esophagus is then opened over the mass and extended a few centimeters proximal and distal to the lesion. Circumferential mobilization of the esophagus is not needed for right-sided lesions; therefore the mediastinal pleura can be opened directly over the esophagus. Lesions lying within the esophageal wall on the anterior, posterior, or left aspect of the esophagus typically require circumferential mobilization so that the esophagus can be rotated to provide clear visualization of the lesion. In these cases, the mediastinal pleura is opened along its junction with the visceral pleura of the posterior lung and the plane between the pericardium and anterior esophagus is developed.

**Figure 4:**
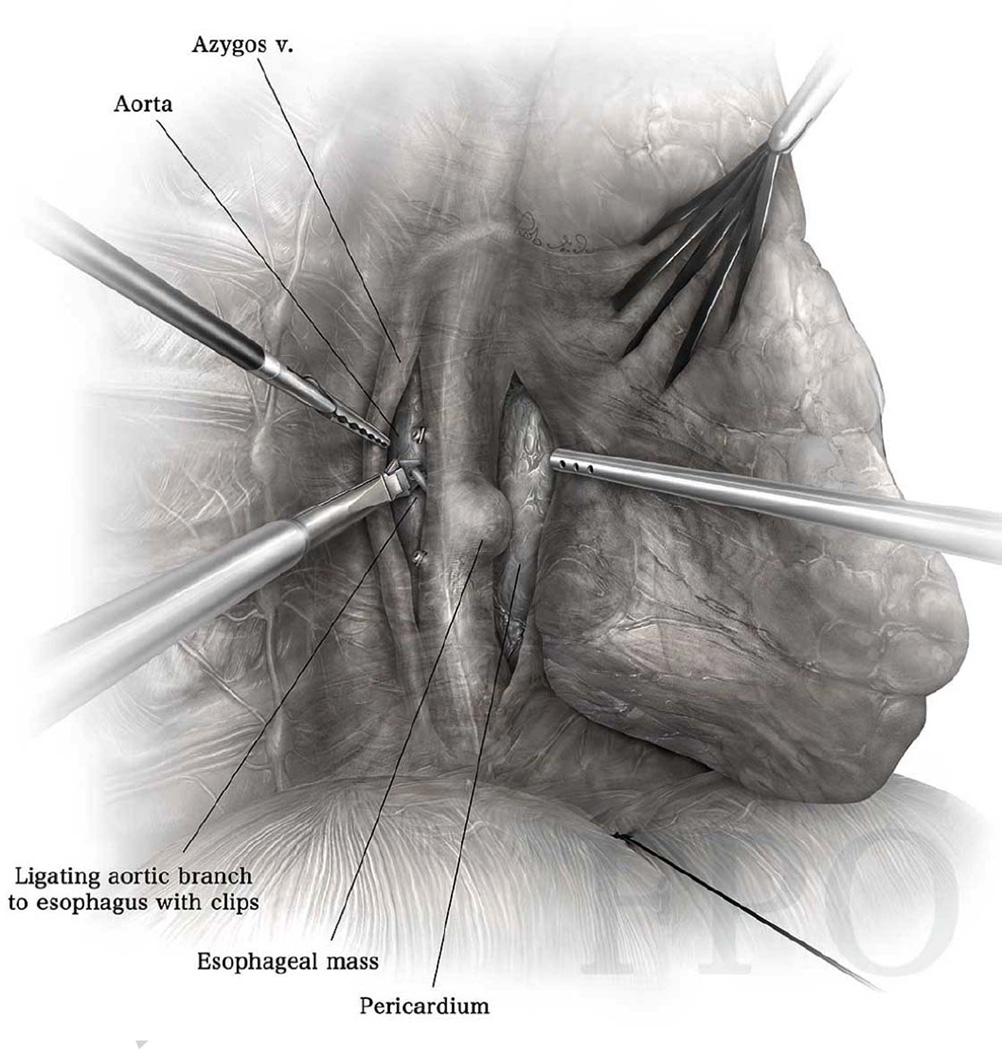
The mediastinal pleura posterior to the esophagus is then opened and the plane along the aorta is developed. Care should be taken to avoid dissection in the fat posteriorly to avoid injury to the thoracic duct. Large aortoesophageal and lymphatic branches are clipped and divided. In contrast to mobilization for an esophagectomy for malignant disease, where periesophageal fat and lymphatic tissue is swept toward the esophagus, dissection is performed directly on the esophagus in benign cases. Dissection is continued along the left aspect of the esophagus until the anterior dissection plane is met. A Penrose drain can then be looped around the esophagus to provide retraction for additional mobilization and exposure.

**Figure 5:**
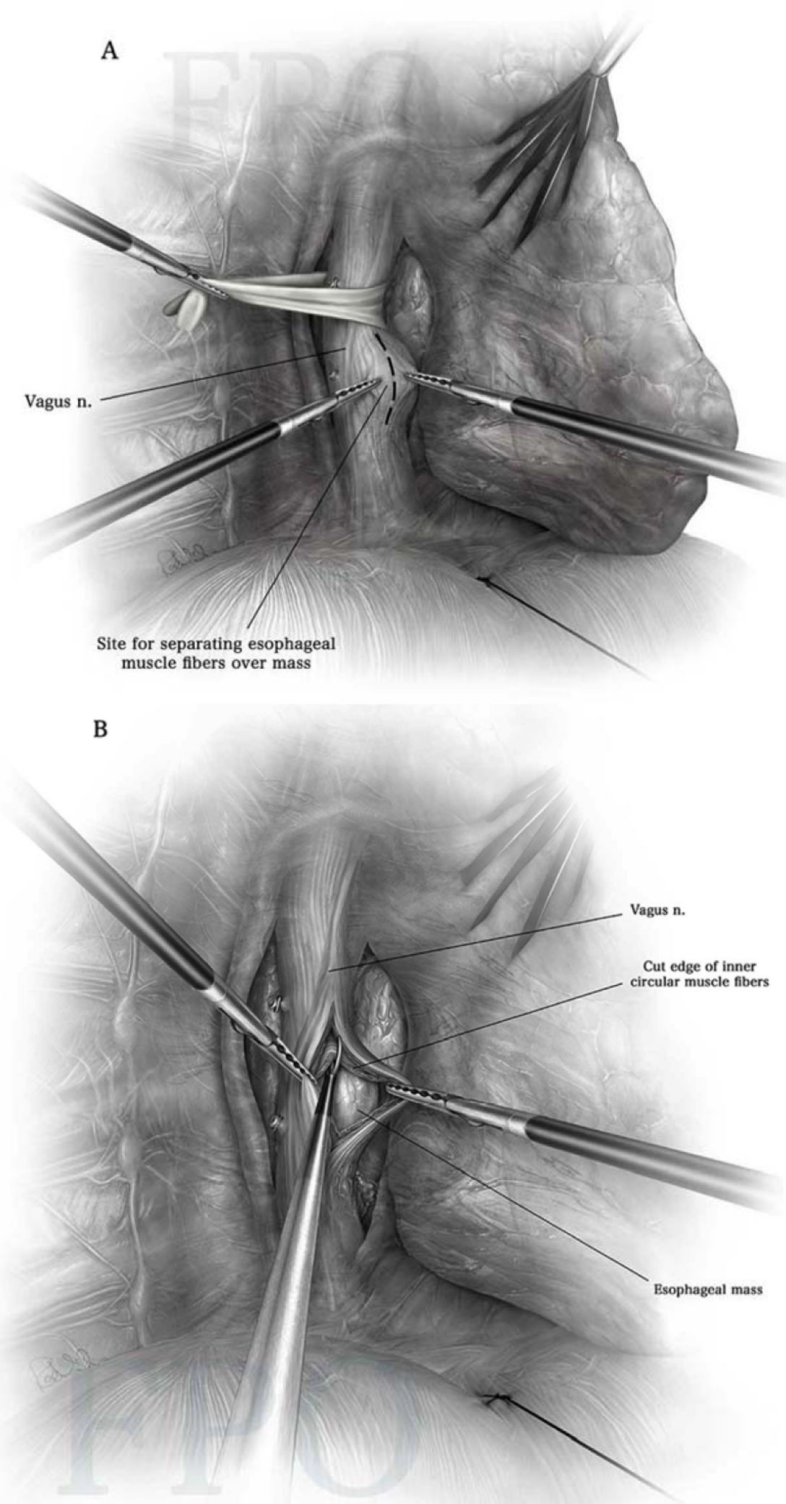
**A&B**. The lesion is then located by direct vision and palpation. If the lesion is difficult to find, intraoperative upper endoscopy may be performed to aid with localization. Passage of a bougie may also help to exaggerate the location of the lesion by displacing it radially. Distraction with either the endoscope or a bougie is highly recommended, as large lesions can cause transluminal protuberance, and the wrong side of the esophagus may be entered. A myotomy is then performed directly over the lesion. The outer longitudinal muscle is separated (not divided) with a blunt pulling technique, hook cautery, or ultrasonic shears. The inner circular muscle fibers are then divided sharply with hook cautery or ultrasonic shears to expose the lesion. Unlike a Heller myotomy where the muscle may be quite thickened, the muscle fibers overlying the lesion are often attenuated and separate easily. The myotomy is carried proximally and distally only as far as needed to identify the borders of the mass. The vagus nerves should be identified and preserved. The vagus nerve can be mobilized and swept away to allow for extension of the myotomy beneath the path of the nerve if necessary.

**Figure 6:**
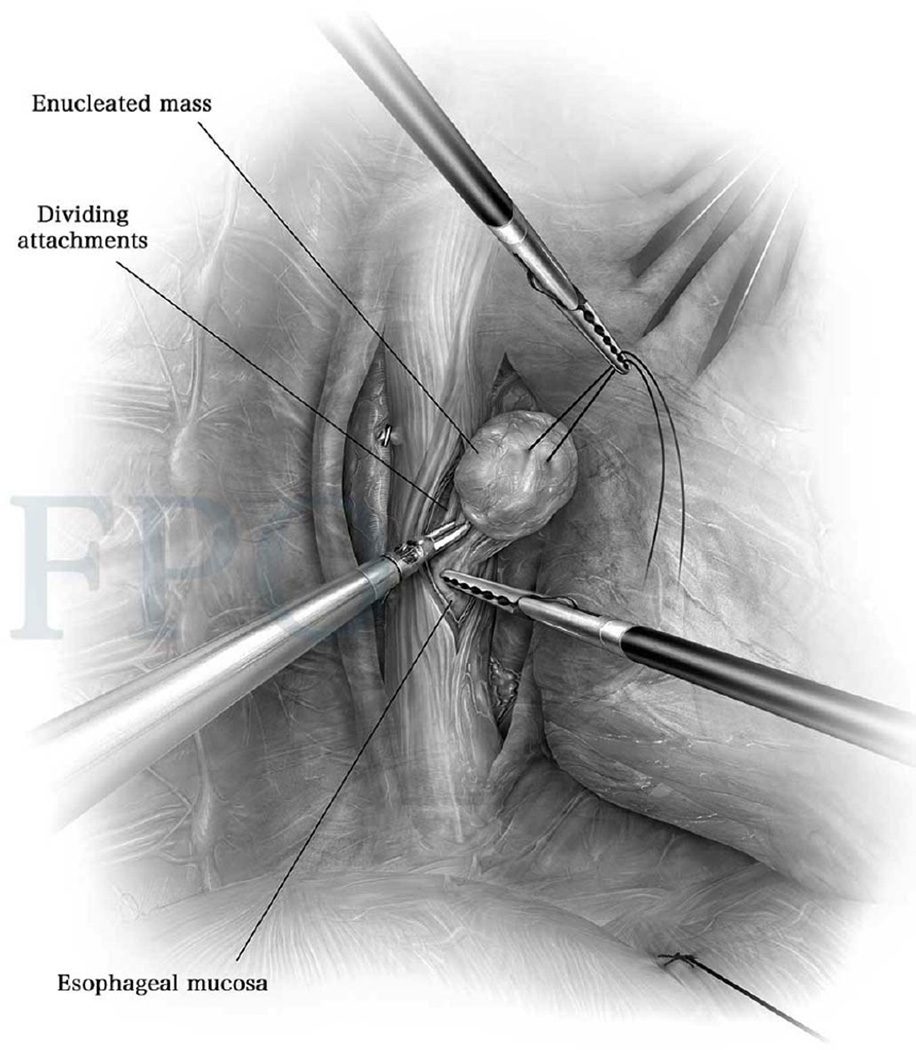
The lesion is then enucleated, keeping dissection directly on the mass. The muscle on each side of the myotomy is dissected away from the mass circumferentially. As the dissection is carried along the deep aspect of the lesion, the mucosal layer is exposed; care must be taken to avoid thermal injury to the underlying mucosa or frank penetration into the lumen of the esophagus. A suture may be placed into the lesion to provide additional retraction, lifting the mass away from the underlying mucosa. An endoscopic retrieval bag is then used to remove the mass from the chest. A retraction stitch should not be placed in cystic lesions, which are typically thin-walled. In these cases, meticulous dissection and gentle retraction are required to avoid rupturing the cyst, as rupture can make complete resection more difficult.

**Figure 7:**
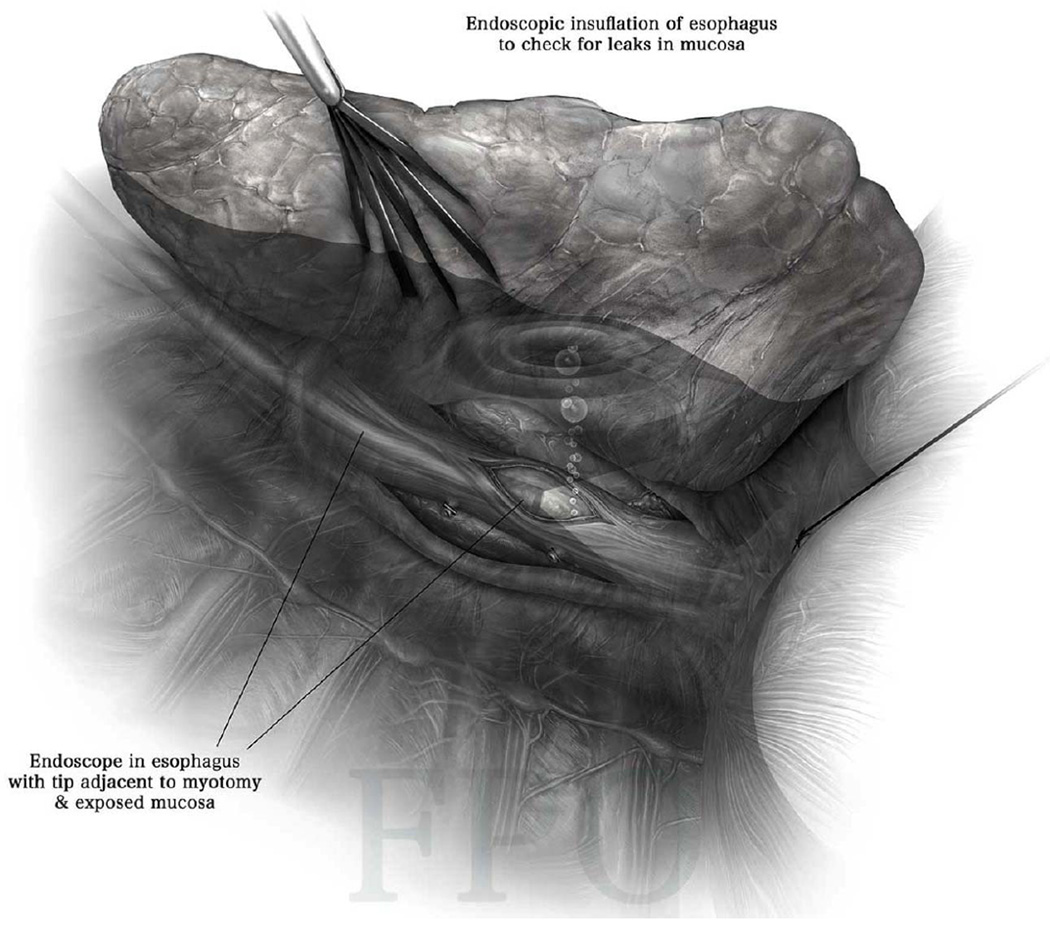
Once the mass has been enucleated, intraoperative upper endoscopy is performed to check for violation of the esophageal mucosa. The esophagus is submerged under water and the endoscope is used to insufflate air into the esophagus, causing the mucosa to bulge at the myotomy site. This allows the surgeon to identify any sites of perforation, as air bubbles will be seen escaping from the lumen at the site of mucosal injury. Mucosal perforation is uncommon, but intraoperative assessment is mandatory and, when identified, is easily repaired with minimal risk of postoperative complication. Most mucosal injuries can be repaired with simple interrupted 2-0 or 3-0 absorbable suture. Larger defects can be closed with suture or a linear endoscopic stapler using a vascular load. A 54-French bougie should be carefully passed under direct thoracoscopic vision prior to attempting stapled repair in order to avoid excessive narrowing of the lumen.

**Figure 8:**
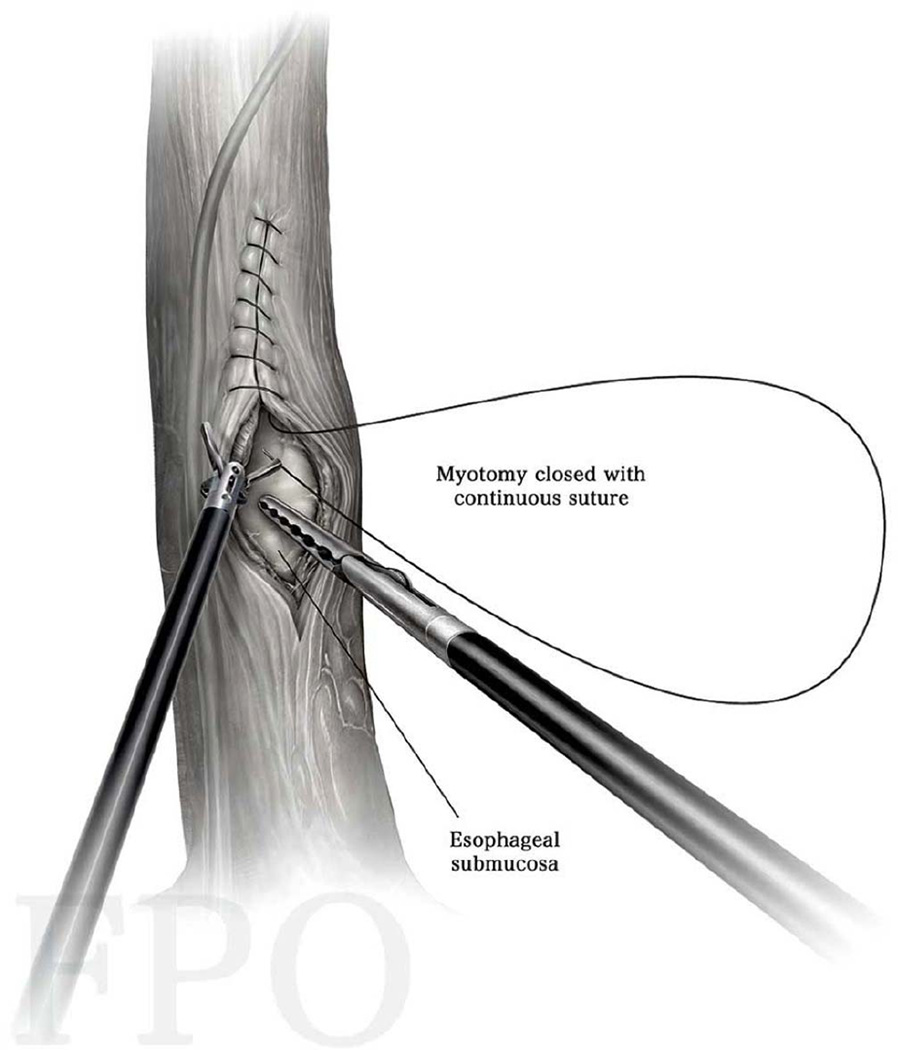
The myotomy is then closed with a running or interrupted 2-0 absorbable suture, making sure to take full-thickness bites of the muscularis propria. A single chest tube or Blake drain is then placed posterior to the lung and directed toward the apex. In cases where a mucosal perforation was repaired, the authors prefer to leave a #10 Jackson-Pratt closed-suction drain adjacent to the myotomy closure to monitor for a postoperative esophageal leak.

**Figure 9:**
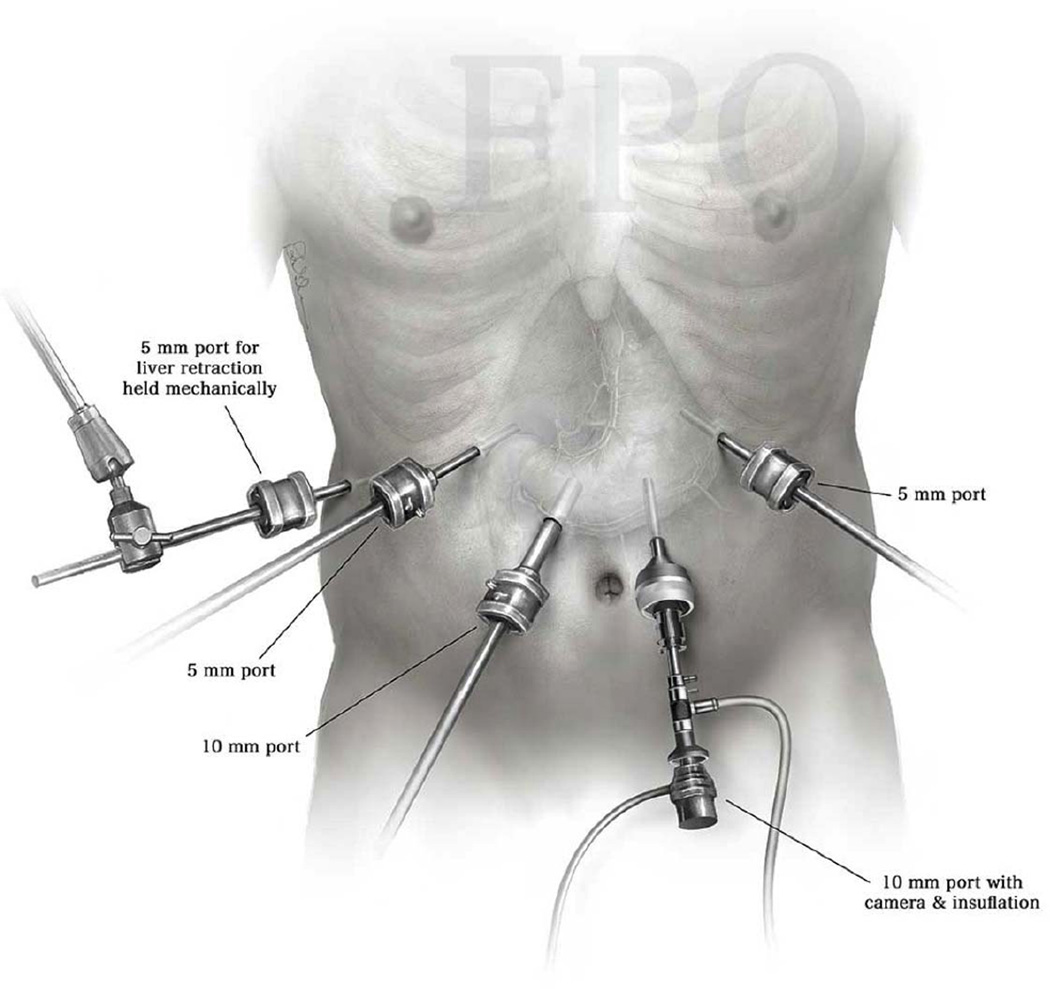
Lesions located in the distal 5 cm of the esophagus or at the gastroesophageal junction are more easily approached laparoscopically. Depending on the surgeon’s preference, the patient is positioned supine or in modified lithotomy. In the authors’ practice, the patient is supine; the operating surgeon stands on the right side of the table and the assistant on the left. A footboard facilitates steep reverse Trendelenberg positioning. To avoid potential injury to any abdominal viscera or organs, the initial port is placed via an open cut-down technique. The 10 mm port is placed two-thirds the distance from the xiphoid to the umbilicus and slightly off the midline in the right upper quadrant for the surgeon’s right-handed working port. The remaining 4 ports are then placed under direct laparoscopic vision. Another 5 or 10 mm port is placed in the left upper quadrant about one hands-breadth away from the initial port and will become the camera port. Bilateral 5 mm ports are placed just below the costal margin, approximately in the mid-clavicular line, for the surgeon’s left-hand and the assistant’s right hand. A 5 mm port in the right flank is used for the liver retractor.

**Figure 10:**
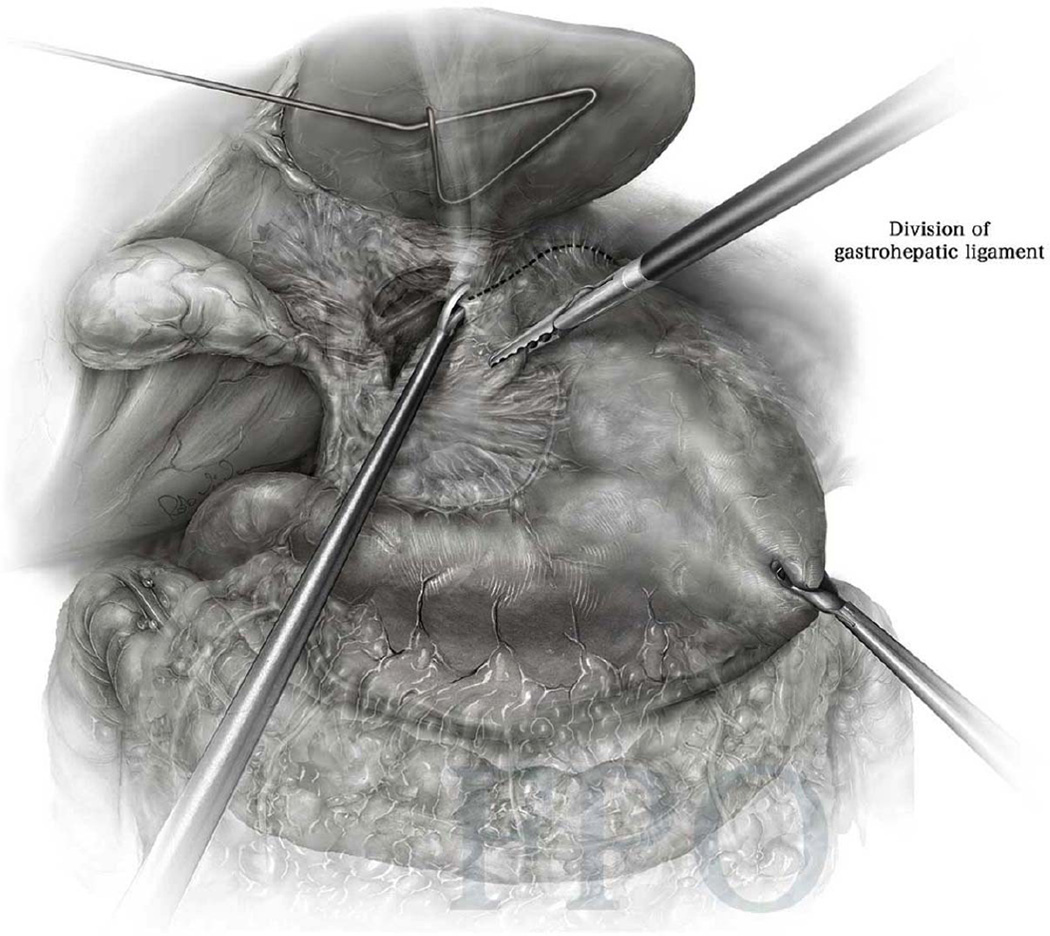
The abdomen is insufflated with carbon dioxide to a pressure of 15 mmHg. The left lobe of the liver is retracted and the patient is placed in steep reverse Trendelenberg position. The gastrohepatic ligament or lesser omentum is opened and dissection is carried from right to left to the angle of His across the arch of the crus, freeing the esophageal fat pat away from the diaphragm.

**Figure 11:**
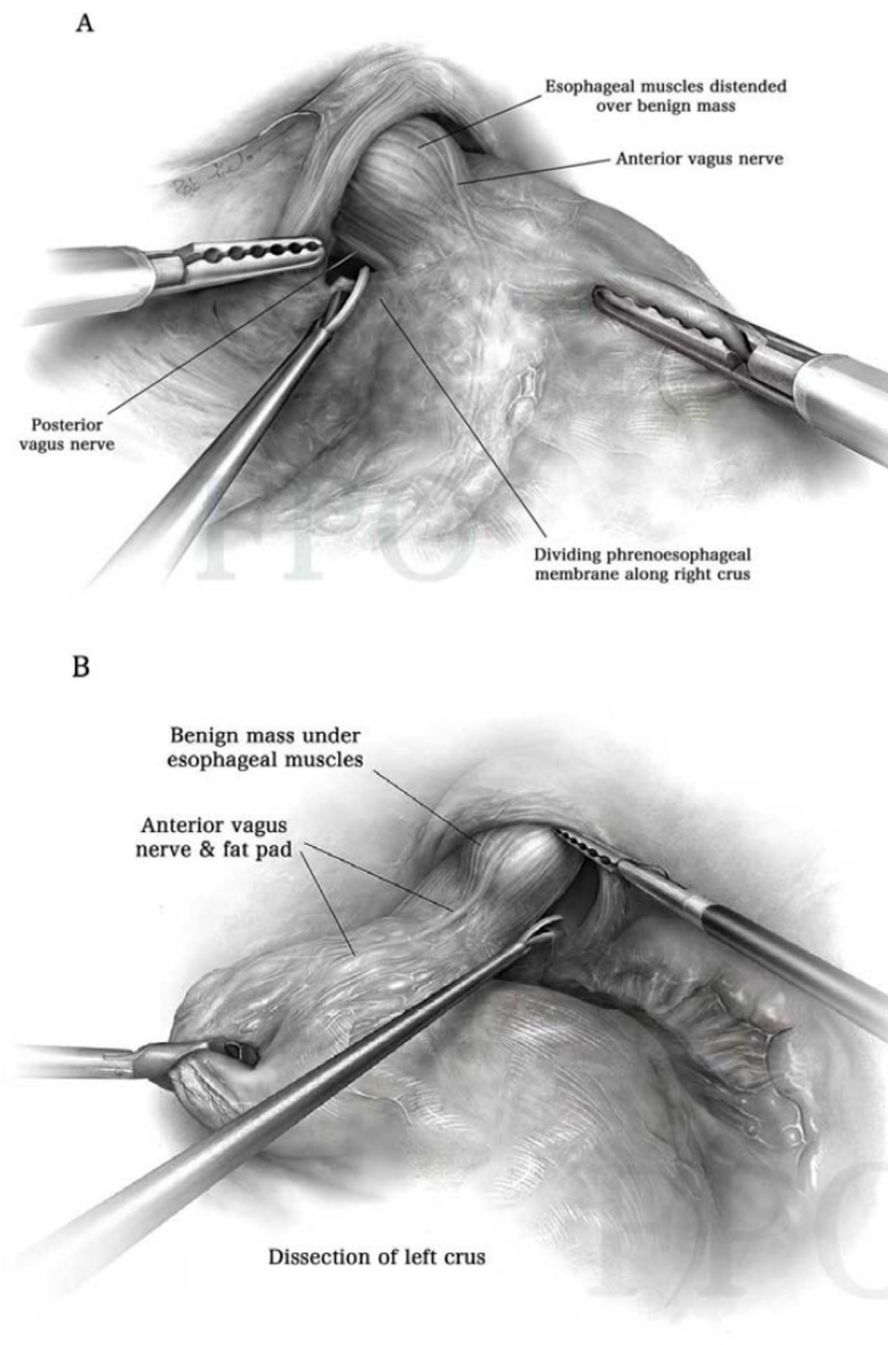
**A&B**. The phrenoesophageal membrane is then opened along the right crus to begin mobilizing the distal esophagus. Care is taken to preserve the peritoneal lining of the crus to allow for better purchase of tissue should the right and left crus need to be reapproximated later. The posterior vagus nerve is identified and preserved. Dissection is carried along the arch of the crus and then down the left crus, taking care to identify and preserve the anterior vagus nerve. The intrathoracic esophagus is then mobilized by dividing the areolar attachments within the mediastinum along the pericardium and bilateral pleura. Dissection is carried proximally only so far as needed to identify the lesion. Lesions located anteriorly do not require circumferential mobilization and retroesophageal attachments should be preserved. Lesions located within the wall of the esophagus posteriorly will require full mobilization as rotation of the esophagus will be necessary to perform myotomy and enucleation. In these cases, the phrenoesophageal membrane along the base of the crus is opened and the plane along the aorta is developed. Division of the short gastric vessels and gastrosplenic attachments is also necessary in this situation.

**Figure 12:**
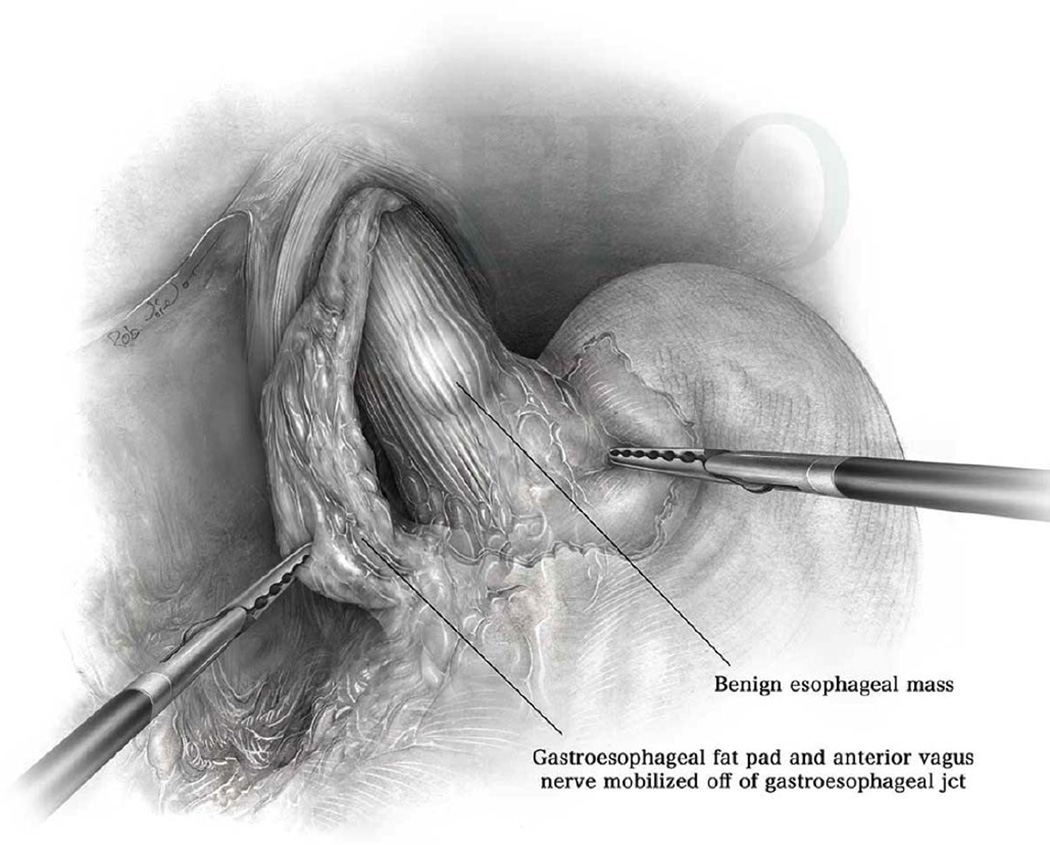
For anterior lesions, the esophageal fat pad is mobilized from the patient’s left to right, sweeping the distal anterior vagus nerve along with the fat pad toward the caudate lobe. The posterior vagus nerve may need to be mobilized for more posterior lesions to facilitate myotomy and enucleation (not shown).

**Figure 13:**
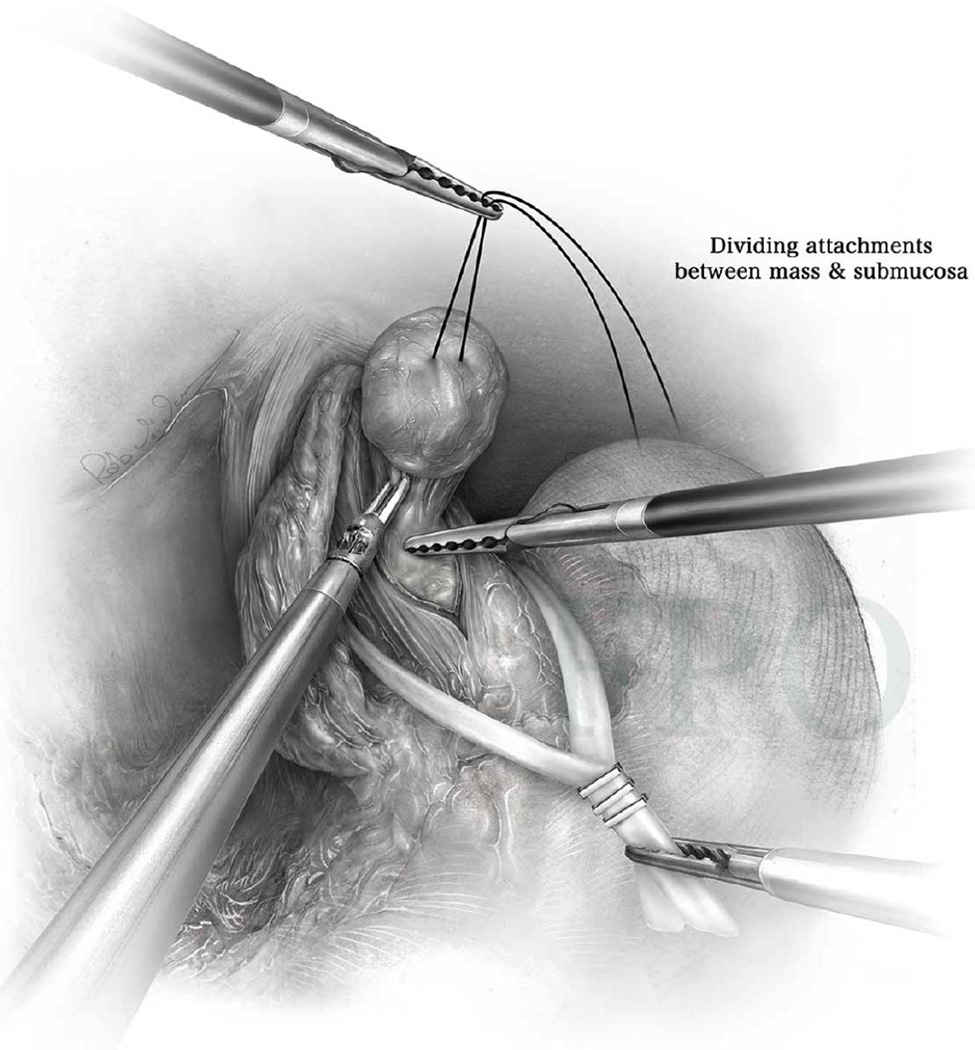
The myotomy and enucleation of the lesion is then carried out as previously described. A Penrose drain may be looped around the distal esophagus to provide retraction inferiorly, bringing the area of interest into better view. Lesions that cannot be adequately visualized with this maneuver may be better approached from the chest. After evaluating for mucosal injury/perforation, the myotomy is closed as previously described. If circumferential mobilization of the distal esophagus was required or the hiatus was widened as a result of extensive intrathoracic mobilization, reapproximation of the crus posteriorly and a Nissen fundoplication is recommended. In cases where minimal hiatal dissection has been performed and posterior attachments remain intact, one may chose to omit a fundoplication or perform a partial anterior (Dor) fundoplication.
